# The length of a piece of string: Where the whole is more than the sum of its constituent parts

**DOI:** 10.1007/s00426-026-02336-z

**Published:** 2026-06-29

**Authors:** Frédéric Devinck, Olivier Le Bohec, Alexandra M. Johnstone, Arash Sahraie

**Affiliations:** 1 Département de Psychologie, Université Rennes 2, Rennes, 35000 France; 2 Rowett Institute, School of Medicine, Medical Sciences and Nutrition, University of Aberdeen, Aberdeen, AB24 3FX UK; 3 Department of Psychology, School of Psychology, University of Aberdeen, William Guild Building, Aberdeen, AB24 3FX UK

**Keywords:** Size perception, Magnitude estimation, Length perception

## Abstract

Determination of the magnitude or size of objects is crucial in many activities of daily living. Here we provide psychophysical evidence across multiple experiments that judgements for accumulated length of fragmented lines are non-veridical, with a larger number of fragments leading to a larger reduction in perceived size. The reported reduction in the size of fragmented stimuli is not a result of stimulus bias or frequency of observations and reflects an inherent perceptual bias. We have extended the findings to real objects in laboratory conditions. Whilst the discrimination threshold for two intact lines remained similar to those reported previously, the reduction in perceived length with fragmentation persisted. Finally, we gathered behavioral data on a real-life task of matching the length of a strand of cooked spaghetti using spaghetti pieces of varying sizes. Participants used progressively larger amounts of spaghetti to match reference length when selecting from smaller cut pieces. The reduction in the perceived magnitude of small segments remained evident when participants decided on the portion size of cooked spaghetti. The converging evidence from multiple investigations demonstrates the robustness of the observed reduction in perceived size with fragmentation.

**Supplementary Information:**

The online version contains supplementary material available at https://doi.org/10.1007/s00426-026-02336-z.

## Public significance statement

Perception is the psychological interpretation of sensory signals that encode the physical properties of stimuli. Some of these interpretations such as perceived size change depending on the scene context. In a series of experiments, we demonstrate that segmentation of line stimuli leads to significant underestimation of cumulative magnitude or line size. For instance, a line object fragmented into 10 pieces is perceived to be 60% of the size of the intact line.

## The length of a piece of string: where the whole is more than the sum of its constituent parts

“How long is a piece of string?” is equivalent to asking how much of something there is. The obsession of humans with measurement of quantity/magnitude probably goes back to the beginning of civilization (Cooperrider & Gentner, 2019) with the earliest documented units of measurement dating as far back as 5–6 millennia (Kaaronen et al., 2023). Physical units allow accurate replication and aid mutual understanding in transactional interactions. In the absence of a measuring device, we rely on sensory inputs to assess the stimulus magnitude. There are two ways of tackling the question of stimulus magnitude such as the length of a string. One is a relative measure, that is, whether it is the same or different from (i.e., greater or less than) the length of a reference string. The other would be to determine or guess the absolute amount/magnitude. The former is relatively simple, and the methods of such comparisons were formalised in the foundations of Experimental Psychology and have been applied to all sensory inputs (Teghtsoonian, 1971). Our sensory systems are highly tuned to identifying a change in input or performing direct comparisons, leading to small just noticeable differences. The latter estimations are much harder to make.

Sensitivity of line length judgement, was reported by Weber (1849, see translation (Weber, 1996) and Fechner (Fechner, 1860), and has been extended to conditions of simultaneous and delayed matching (Ono, 1967; Stevens & Galanter, 1957; Teghtsoonian, 1965); and has been studied in other mammals (Wartzok & Ray, 1976). Consistently, the findings show high sensitivity of the visual system in extracting information on the line length such that a difference of 1.5-3% over a wide range of sizes is reliably detected (Ono, 1967). There is another way of forming a line stimulus and that is by defining its endpoints. The perception of such imaginary lines would differ from a physical line in that for fixed endpoints, the perceived length of the imaginary line would change depending on the number of objects placed in the intervening space with more objects resulting in a larger perceived length. This phenomenon is called the Oppel-Kundt illusion (Luckiesh, 1922), and has a counter-intuitive turn, in that the presence of only one item along the imaginary line, would lead to being perceived as shorter than had it been an empty space (Mikellidou & Thompson, 2014). In other words, dividing an imaginary line into two segments would make the total perceived as shorter in length.

The ability to reliably report the size difference between two 2D surfaces is more complex. It is proposed that area perception relies on the precision of two orthogonal length estimates (such as width and height) (Morgan, 2005) when the object aspect ratio is close to unity (Morgan, 2005; Nachmias, 2008), but the observed thresholds deviate from the estimates for ellipses and curved surfaces (Nachmias, 2008, 2011). Although the relationship between the perceived and mathematically determined length of intact lines is linear, it diverges for surfaces and is better described by a power law function (Teghtsoonian, 1965) with exponent of around 0.8. It is also proposed that the summation of the magnitude estimates along the orthogonal lines, rather than their multiplication, may more accurately reflect the perceived size (Carbon, 2016; Yousif & Keil, 2019, 2021). This approach could also be extended to the perceived sum of multiple surfaces (Carbon, 2016; Yousif & Keil, 2021), and even to volume perception (Bennette et al., 2021), although at least some of these findings have been disputed (Park, 2022; Yousif & Keil, 2022). In common with all the above observations and proposals, there is the need for accurate multiple length estimation as well as estimation of their accumulated length.

For non-continuous lines, the integration of contours within a range of spatio-temporal window allows integration across gaps (Hess et al., 2001). These properties are thought to underline our perception of partially occluded objects, facilitating everyday object perception (Spillmann, 2009). Likewise, integrating similarly oriented (colinear), spatially distinct Gabor patches within randomly distributed Gabors has been found to be highly efficient in both humans (Beck et al., 1989) and macaque studies (Mandon & Kreiter, 2005). This collinearity facilitates bottom-up spatial integrations of contours at the neuronal level (Schmidt et al., 1997), probably representing the neuronal correlates of good continuation (Polat & Sagi, 1994). Both the contour’s presence and its shape can be discriminated even at brief masked stimulus durations of 100-300ms (Hess et al., 2001) or in presentation duration as short as 30-60ms in macaques (Mandon & Kreiter, 2005) indicating the efficiency of bottom-up processes.

The findings from studies described above all rely on investigations that involve presentation of basic visual stimuli (e.g., luminance defined Gabor patches) on computer displays, whilst the findings are interpreted as fundamentals of visual performance in natural settings. Although contribution of studies using such simplified 2D representations is valuable, there is now growing evidence for differences in their processing compared to real life stimuli. These extend, but are not limited, to real stimuli being better remembered (Snow et al., 2014), modulating attention (Gomez et al., 2018) or better recognized (Holler et al., 2019). These enhanced abilities are termed real-object advantage (Snow & Culham, 2021). Therefore, ecological experiments are useful in testing the hypothesis developed under controlled conditions, under more variable natural settings.

Here we have outlined ample psychophysical evidence showing great precision in detection of size differences between nearly identical continuous lines. Similar precision has been reported when comparing one dimension of a 2D surface such as diameter of circles. There is also great efficiency in the visual system to detect non-continuous contours and integrate their length even in noisy background. These efficiencies also apply to imaginary lines with the caveat that segmentation of imaginary lines leads to changes in perceived space depending on number of segmentations. Overall, given this evidence, it is reasonable to hypothesize the presence of similar thresholds for size estimation/discrimination of multiple discrete segments as those for single continuous lines.

We report below on the novel and surprising findings from eight experiments that map out the characteristics of summative length estimation, which underpin the working of proposed mechanisms allowing line size judgement. By combining controlled computer-based psychophysical tasks, laboratory based observations and real life tasks, we demonstrate the relevance of the findings to everyday interactions with our environment.

## Experiment 1: Effect of fragmentation on perceived length

The aim of this experiment was to obtain sensitivity and bias in size discrimination between an intact reference line and fragmented lines. To achieve this, we set out to obtain the full psychometric function for the comparison of tests with reference stimuli. Prior to the conduct of experiments outlined in this manuscript, we did not know the range of stimulus parameters needed for a reliable probit fit, the effect size, or the sample size needed, therefore a pilot investigation was conducted.

### Pilot investigation to determine sample and effect size, and stimulus parameters

In a pilot experiment, participants compared the total length of fragmented lines with that of an intact reference length, where the test stimuli were either intact lines or fragmented into 2, 3, 4 or 5 segments to form 5 experimental conditions. For each condition, 8 different length sizes were presented, with 24 observations/trials per length (192 trials per condition). Summary data shown in supplementary material evidenced a systematic size length underestimation with increasing fragmentation. Confidence intervals of the mean point of subjective equality for all conditions were determined. The pilot investigation showed non-overlapping confidence intervals between all conditions, except between 4&5 segments, with large effect sizes (all dz > 2.77), therefore the chosen sample size of 18 provided adequate power to identify the perceived size underestimation with fragmentation. The proximity of PSEs for 4&5 segments might also point to the possibility that thresholds for PSEs could be limited to having a small number of fragments, hence the number of fragments were also extended to explore this possibility. Having added a 10 segments condition, the 4 segments condition was removed to keep the duration of experiment the same approximately 40 min). A full description of the pilot, group average and individual participant data are all documented in the supplementary material.

## Methods

Experiments 1, 2 A, 2B, 3 and 4 were conducted online and experiments 5, 6 A and 6B were conducted in person under laboratory conditions. The participant recruitment section outlined below, and calibration procedures apply to all online experiments.

### Participants

For all online studies, adult participants were recruited through Testable Minds (testable.org) with the only criteria being age > 18 and having self-reported normal or corrected to normal vision and a Testable reliability score of > 90%. Testable confirms age and identity prior to taking part in the experiment. Participants were naive to the purpose of the experiments and placed on a Testable excluded panel to limit participation in more than one experiment. For all online studies, participants were paid at a rate of $12 per hour. Testable uses facial information to confirm the identity of participant panel members prior to taking part in the experiment to ensure the data is not generated by AI.

20 participants (9 F, *M*_age_ = 39.1, *SD* = 14.50, *range* = 22–62) took part in experiment 1.

Prior to any data collection, all protocols, inclusion/exclusion criteria and the planned data analysis, including pilot investigations, were documented and preregistered on the Open Science Forum (https://osf.io/xubpm/). Ethical permissions were obtained for the conduct of the experiments outlined from the psychology research ethics review boards at both University of Aberdeen (PEC4461402) and Université de Rennes 2. All participants indicated their informed consent before progressing to the online experiments. Data was collected between June 2024 and September 2025. No identifying data, except age and sex were retained for the participants in all the experiments reported in this manuscript.

Both vigilance and accuracy were essential for the experiments outlined in this manuscript. All experiments included both catch trials and measures of reaction time. Those participants who responded with reaction times faster than 200ms or time-out trials, comprising more than 8% of the trials were excluded, as well those who failed 20% (5 out of 24) or higher number of catch trials. In the pilot investigation, the test length needed to ensure a full psychometric function when comparing the length of two lines were established and the presence of this psychometric function was used as a measure of complying with task instruction, so those who could not make accurate judgement about the size of two lines, were therefore excluded from the studies requiring the judgement on multiple segments.

### Stimuli

Stimuli were in .jpg format developed using GIMP (version 2.10.38). These were 800 × 600 pixels (width x height) images with reference and test lines being presented such that their midpoints were within a 100 × 100 pixels box placed at ± 200 pixels (right and left) of the center. The position of line segments was randomized within the box with the constraint that lines could not touch or intersect. The line stimuli were RGB color 0,0,0 presented on a grey background (RGB 128,128,128). Fixation was a small black cross (16 × 16 pixels) at the center of the screen that was displayed for 500ms prior to the stimulus presentation which was 250ms in duration. Durations were verified in each trial by counting and recording the number of frames that a stimulus was presented. The response window was 2 s. The inter-trial time was set to 800ms. Participants could only perform the task on a computer monitor (not on portable devices) and respond using left/right arrow keys to indicate the side of the screen that contained more length. The refresh rate, screen resolution, and set up calibration parameters of the participant’s device were recorded.

Lines, in intact line comparisons (comparison of a reference and one test line) always had mirroring orientations with respect to the vertical meridian along ± 15 or ± 30 degrees. This together with random jitter on the horizontal and vertical coordinates of their center points were used to minimize size judgements being based on stimuli end point positions. Reference was always 200 pixels in length and the 8 test lengths for single line test stimuli were 160,180, 190, 195, 205, 210, 220 & 240 pixels.

Two segment tests: the reference target had one of 4 possible orientations (± 15, ± 30) degrees with respect to the vertical meridian, with each of the two test segments being presented one at the same and another at mirror imaged orientation. The 8 test total lengths were 180, 200, 220, 240, 260, 280, 300, 320 pixels. The partitioning of the two segments in each trial was determined by generating a set of random fractions between (60 − 40) % and (90–10) % of the total length generated prior to the construction of stimuli.

3 segment tests: The three-segment condition was utilized exclusively during the initial pilot investigation and in Experiment 2 A; it was not included in the design of Experiment 1. The 8 total lengths for 3 segment conditions were 180, 200, 220, 240, 260, 280, 300 & 320 pixels. We generated 12 images with reference on the left of the fixation, for each of the 8 lengths, with one segment at 4 of the orientations being set to either 80, 70, 60, 50, and 40% of the total length with the remaining 2 sharing the remaining length in multiples of 10% of the total length. Over the 12 images, the incidence of segments having the same or different orientation to that of the reference were equated. The images were mirrored to generate 12 additional images with references line being presented on the right-hand side of the screen.

5 segment tests: the 8 total lengths for 5 segment conditions were 180, 220, 260, 300, 320, 340, 360 & 400 pixels. A line segment could be set to either 50, 40, 30, 20 or 10% of the total length and presented either at the orientation of the reference or at its mirror image orientation. This led to a possible 12 combinations of segment sizes, and they were placed on the left or right of the fixation to form 24 trials for a given total length (all stimuli can be downloaded from the Open Science Forum page https://osf.io/xubpm/).

10 segment tests: the 8 total lengths for 10 segment stimulus were set to 180, 220, 260, 300, 340, 360, 400 & 440 pixels. Each segment was randomly set to between 8 and 12% of the total length with their accumulated length equating the total length for the condition stated. In each image, 5 segments had the same orientation as the reference and the remaining were set to the mirroring orientation with respect to the vertical meridian.

### Procedures

Prior to the start of the experiments, Testable calibration routine was conducted where a yellow horizontal line was placed on the screen on the full screen mode. Participants were asked to adjust the width of the line to that of the width of a standard credit card (85 mm). This calibration factor was applied to presentation of all stimuli to ensure equal stimulus sizes across screens where a 200 pixels reference line had a length of 40 mm, subtending a visual angle 4.0° at 57 cm viewing distance.

All stimuli were presented in random order. There were 24 catch trials, where only the reference was presented. For these, the participants were instructed to identify the side that the line was presented. Timed out trials and those with reaction times below 200ms were excluded.

The instruction to participants was to treat the line segments as pieces of gold and the participants’ task was to make a choice between either the intact line, or the collection of line segments that would maximize their amount of gold. 10 practice trials were conducted prior to the body of experiment, and 4 pauses were introduced to avoid fatigue, approximately every 200 trials. The instructions were to resume the experiment after 1 min pause.

## Results

Data were analyzed using Generalized Linear Mixed Models (GLMM) approach which better accounts for between-observer variability through random effects, yielding more stable population level estimates (Knoblauch & Maloney, 2012; Moscatelli et al., 2012). All analyses were performed using the open-source software R (R Core Team, 2025) with functions from the *Psyphy* (Knoblauch, 2023) the *lme4* (Bates et al., 2026) and the *MixedPsy* (Moscatelli & Balestrucci, 2025) packages.

Across participants, the number of trials on which the test length was reported as greater than the reference was modeled using a GLMM with a probit link. The number of “test longer” responses out of the total number of trials was modeled as a function of stimulus length, line segment condition, and their interaction as fixed effects, with by-observer random intercepts and slopes for length. The point of subjective equality (PSE) was then extracted from the fitted model using the delta method (Moscatelli et al., 2012) and converted back to pixel units. A representative psychometric function was displayed by the mean of a General Linear Model (GLM).

**Fig. 1:**
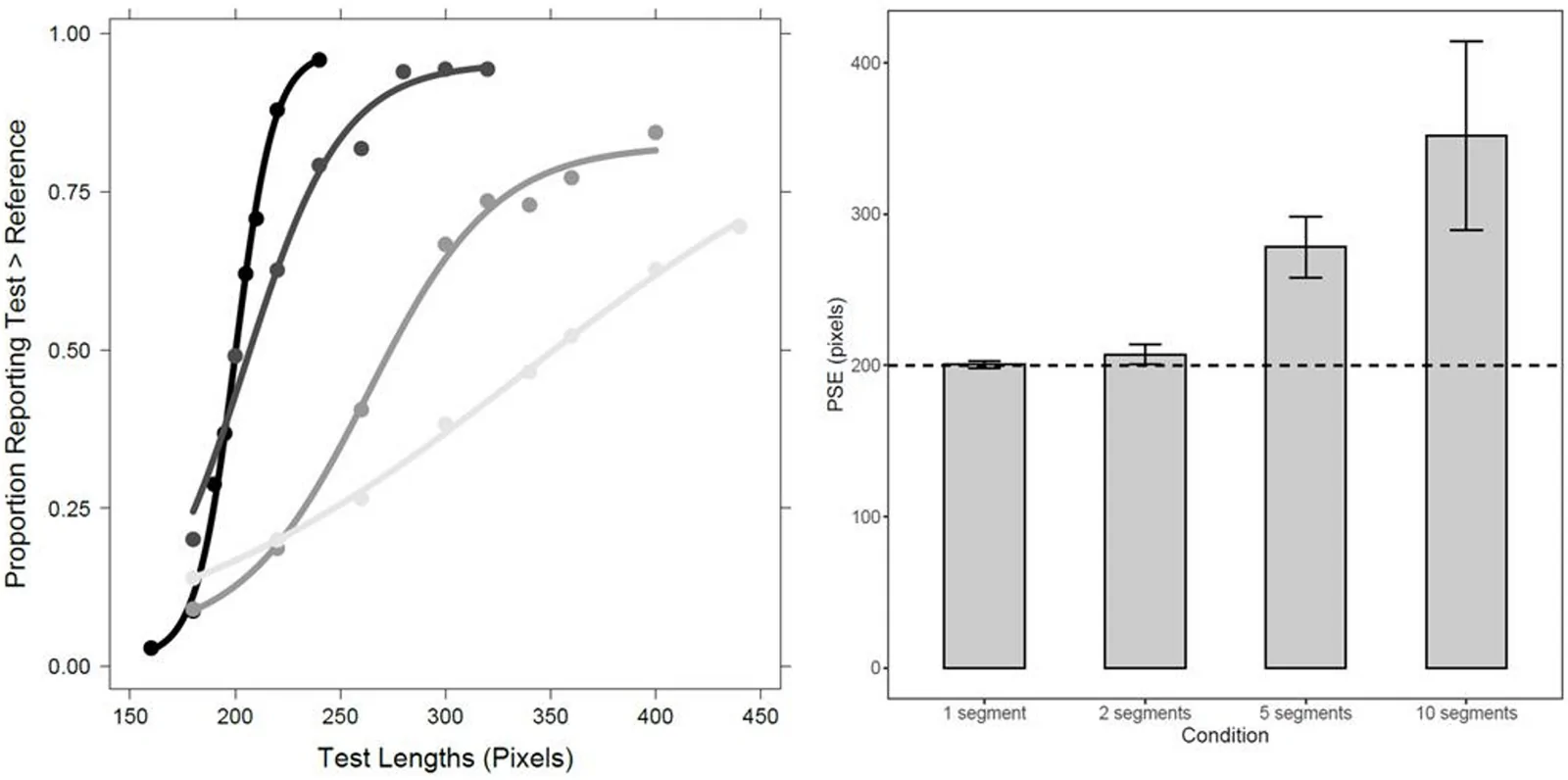
The incidence of reporting the test to be greater than a reference for the 4 experimental conditions forms the psychometric functions shown on the left panel. From left to right, these functions are for 1 (black), 2 (dark grey), 5 (middle grey) and 10 (light grey) line segments averaged for all participants (200 pixels reference line). The average PSEs are plotted on the right panel. Error bars indicate 95% CIs

The probability of reporting that the test was larger than reference for tests comprising of 1 (black), 2 (dark grey), 5 (middle grey) and 10 (light grey) line segments averaged for all participants is shown in Fig. 1.

The slope of the psychometric function is steepest for a single line test and becomes progressively shallower with increasing number of segments. The striking finding is that this reduction of slope is accompanied by a marked rightward shift of the point of subjective equality. Notably, extracting the point of subjective equality (PSE) from the fitted probit functions (plotted on the right panel) leads to thresholds for comparison of a test with a 200 pixels reference line of (*M* = 200.5, *CI* (198.2–202.9)) for a single line; (*M* = 207.2, *CI* (200.7–213.7)) for 2 line segments; (*M* = 278.2, *CI* (258.1–298.3)) for 5 line segments; and (*M* = 351.7, *CI* (289.4–414.0)) for 10 segments. As a percentage of the reference lengths, these thresholds are 0.25%, 3.5%, 39.1% and 75.9% underestimation of length for 1, 2, 5 & 10 segment stimuli. It is a remarkable finding that fragmented stimuli are consistently perceived as smaller in size in contrast to the intact reference length. In other words, fragmentation leads to a systematic reduction in perceived length.

These results were confirmed by the GLMM analysis with a probit link function. The model included stimulus length (standardized), line segment condition (1, 2, 5 and 10 segments), and their interaction as fixed effects, with random intercepts and random slopes for length across observers.

In the single line condition (reference level), the intercept was significantly positive (*Estimat*e = 3.54, *SE* = 0.17, *z* = 21.39, *p*< .001). This indicates that, at the mean stimulus length, observers were more likely to judge the test stimulus as longer than the reference. The slope for length was also significantly positive (*Estimat*e = 4.09, *SE* = 0.15, *z* = 27.65, *p*< .001). This reflects a steep psychometric function and a high discrimination precision in this condition. Compared to the single line segment, the intercepts were significantly reduced for 2 line segments (*Estimat*e = -2.26, *SE* = 0.11, *z* = -20.30, *p*< .001), 5 line segments (*Estimat*e = -3.73, *SE* = 0.11, *z* = -34.02, *p*< .001) and 10 segments (*Estimat*e = -4.16, *SE* = 0.11, *z* = -37.91, *p*< .001). This shift in intercept corresponds to an increase in the PSE, indicating that progressively longer physical lengths were required for the test stimulus (2, 5 and 10 line segments) to be perceived as equal to the reference.

Finally, all interactions between the length and the line segment conditions were significant with increasingly negative estimates for 2 line segments (*Estimat*e = -2.44, *SE* = 0.13, *z* = -18.95, *p*< .001), for 5 line segments (*Estimat*e = -3.04, *SE* = 0.12, *z* = -24.77, *p*< .001) and for 10 segments (*Estimat*e = -3.55, *SE* = 0.12, *z* = -29.39, *p*< .001). This pattern of results indicates progressively shallower psychometric function, resulting in reduced discrimination sensitivity.

## Experiment 2: Effect of stimulus duration

The surprising find of a large and systematic underestimation of length could be attributed to the phenomenon of visual crowding (Bouma, 1970). That is, brief peripheral presentation of several segments may lead to impaired perception of an individual segment surrounded by flankers (Levi, 2008). Although detection of an individual item in crowded scene may not be affected, its identification may be impaired (Whitney & Levi, 2011). Certain features of the crowded scene such as average orientation may be preserved (Parkes et al., 2001), but it is unclear if the object size could be retained. Hence, if individual size information is diminished under crowding conditions, it could contribute to the observed reduced accumulative size information. To test this hypothesis, we conducted two experiments where we changed the experimental conditions, mainly the stimulus duration, allowing for active exploration of items, in order to reduce the potential effect of crowding. Should the same pattern of results persist, the reduction in perceived length cannot be accounted by crowding.

### Procedures

Both experiments 2 A and 2B included the size discrimination of a single target line with a reference line. In experiment 2 A, following the presentation of a fixation point (500ms), we randomly interleaved stimuli of 3 and 5 segments, both shown for either 250ms or 1000ms. The response window was also increased to 2500ms. It was anticipated that the psychometric functions for the short and long duration conditions would deviate from each other with the longer durations shifting more to the left, more resembling the psychometric function for comparison of two intact lines. That is, because the longer presentation time is presumed to allow for longer exploration of stimuli, reducing the effect of crowding. In a variant of this condition (experiment 2B), to encourage stimulus exploration, we removed the fixation point altogether. A new group of participants were instructed to make the size discrimination for 5 and 10 segment stimuli presented for 2500ms with a 3000ms response window. The lack of any fixation point, and long duration would allow ample time for exploration of the scene and response. All the instructions to the participants were the same as those for experiment 1. Number of presentations per condition was the same as in experiment 1 (192 trials).

### Stimuli

All single line, 3, 5 and 10 segment stimuli were the same lengths as those reported in the previous experiment.

### Participants

20 participants (9 F, *M*_age_ = 34.80, *SD* = 12.11, *range* = 20–64) took part in experiment 2 A and 27 participants (12 F, *M*_age_ = 35.33, *SD* = 11.50, *range* = 22–67) in experiment 2B.

## Results

**Fig. 2:**
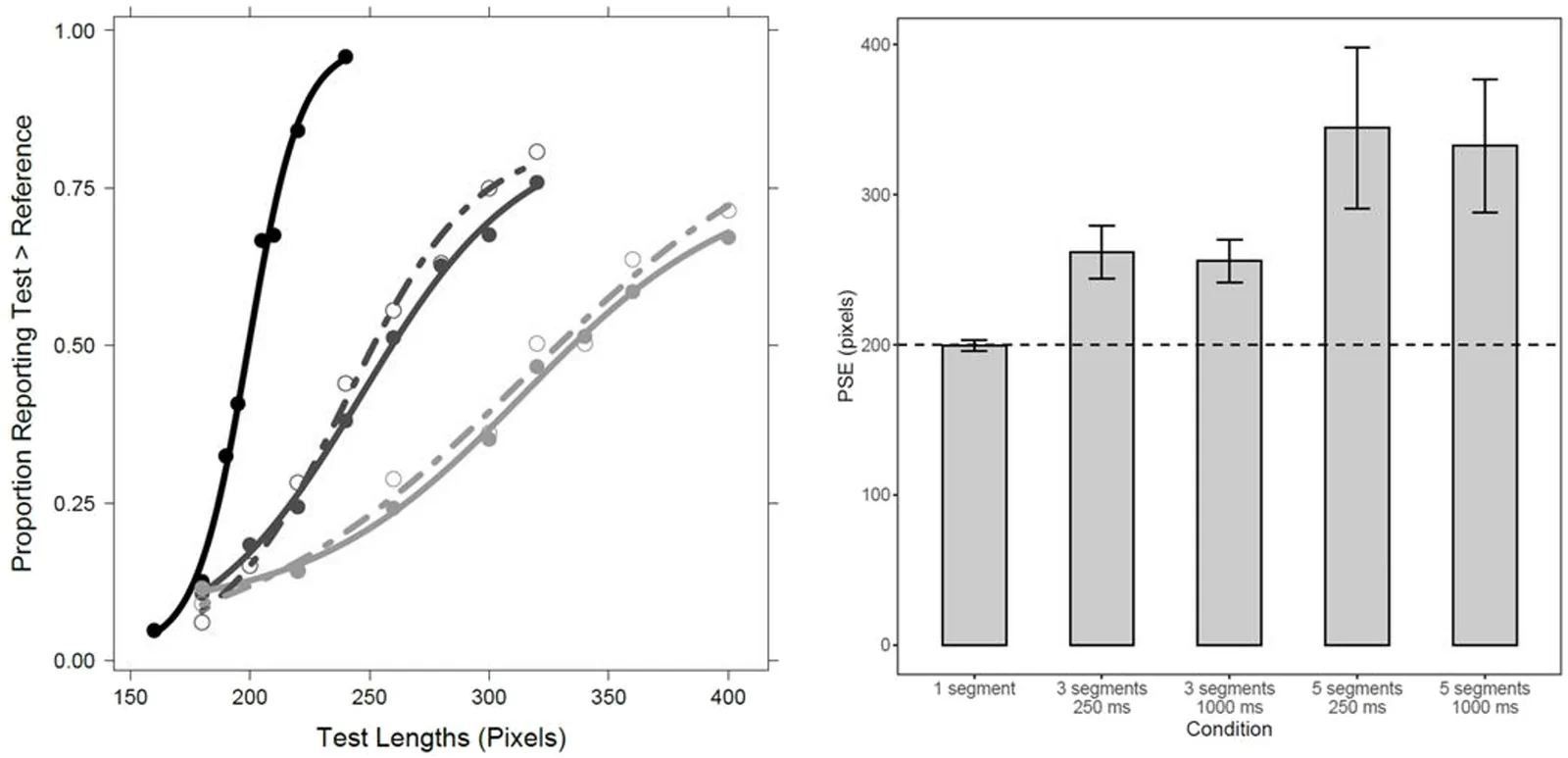
The psychometric functions averaged across participants for reporting the test to be greater than a reference is plotted for 1 (black), 3 (dark grey) and 5 (light grey) segment stimuli at short (solid lines) and long (broken lines) randomly interleaved presentation times, as a function of test length. The right hand panel plots the extracted mean PSEs and their associated 95% CIs

The analysis procedure was identical to that of Experiment 1. On the left panel of Fig. 2, the shorter and longer presentations are depicted as solid and broken lines and dark grey and light grey lines indicate the psychometric functions obtained for 3 and 5 line segments respectively. The right hand panel shows the point of subject equality (PSE) for the five conditions extracted from fitted probit functions. For the comparison of two lines (reference = 200pixels) this was (*M* = 199.5, *CI* (195.9–203.1)) and was similar to the figure in the previous experiment; the values for 3 segments with short and long presentations were (*M*_*short*_ = 261.8 (30.09%), *CI* (244.2–279.4)) and (*M*_*long*_ = 255.9 (27.95%), *CI* (247.7–270.1)); and for 5 segments (*M*_*short*_ = 344.5 (72.25%), *CI* (291.0–398.1)) and (*M*_*long*_ = 332.6 (66.3%), *CI* (288.1–377.1)) indicating overlapping confidence intervals of the means for the two presentation conditions.

These effects were confirmed by the GLMM. In the single line condition, both the intercept and slope were positive and significant (*Estimat*e = 2.95, *SE* = 0.17, *z* = 17.73, *p*< .001; *Estimat*e = 3.21, *SE* = 0.13, *z* = 25.23, *p*< .001). The positive slope reflects a steep psychometric function and high discrimination precision in the reference condition. In comparison with the single line segment, the intercept was significantly reduced in all segmented conditions: 3 line segments presented for 250 ms (*Estimat*e = -3, *SE* = 0.09, *z* = -39.42, *p*< .001), 3 line segments presented for 1000 ms (*Estimat*e = -2.89, *SE* = 0.09, *z* = -31.52, *p*< .001), 5 line segments presented for 250 ms (*Estimat*e = -3.73, *SE* = 0.09, *z* = -39.71, *p*< .001), and 5 line segments presented for 1000 ms (*Estimat*e = -3.70, *SE* = 0.09, *z* = -39.42, *p*< .001). These shifts are consistent with the increase in PSE observed for line segments, indicating that progressively greater physical length were required for the stimuli to be perceived as equal in length to the single line condition. The interactions between stimulus length and line segments were also significant: 3 line segments at 250 ms (*Estimat*e = -2.15, *SE* = 0.10, *z* = -21.61, *p*< .001), 3 line segments at 1000 ms (*Estimat*e = -1.96, *SE* = 0.10, *z* = -19.54, *p*< .001), 5 line segments at 250 ms (*Estimat*e = -2.62, *SE* = 0.10, *z* = -27.19, *p*< .001), and 5 line segments at 1000 ms (*Estimat*e = -2.55, *SE* = 0.10, *z* = -27.19, *p*< .001). When the number of line segments increases, the negative interaction coefficients increase too, indicating that the discrimination sensitivity decreased. In addition, the similarity of the interaction coefficients obtained for the 250 and 1000 ms presentation durations suggests that time presentation had little influence on discrimination sensitivity.

This lack of a marked change in the psychometric functions can indicate that crowding is not the underlying cause of perceived reduction in size. However, in principle a change in participant’s strategy can also explain the results. As short and long time-intervals were interleaved, the participants may have chosen to keep their fixation at the center of the screen, which could in turn have led to crowding of the peripheral targets. Of note is that the PSE for 5 segment stimuli were 72.25 and 66.3% under these conditions, which were much longer than those found in Experiment 1. This difference may also be related to the changes in experimental conditions. Hence, experiment 2B was conducted to address these possibilities and encourage participants to explore the scene.

**Fig. 3:**
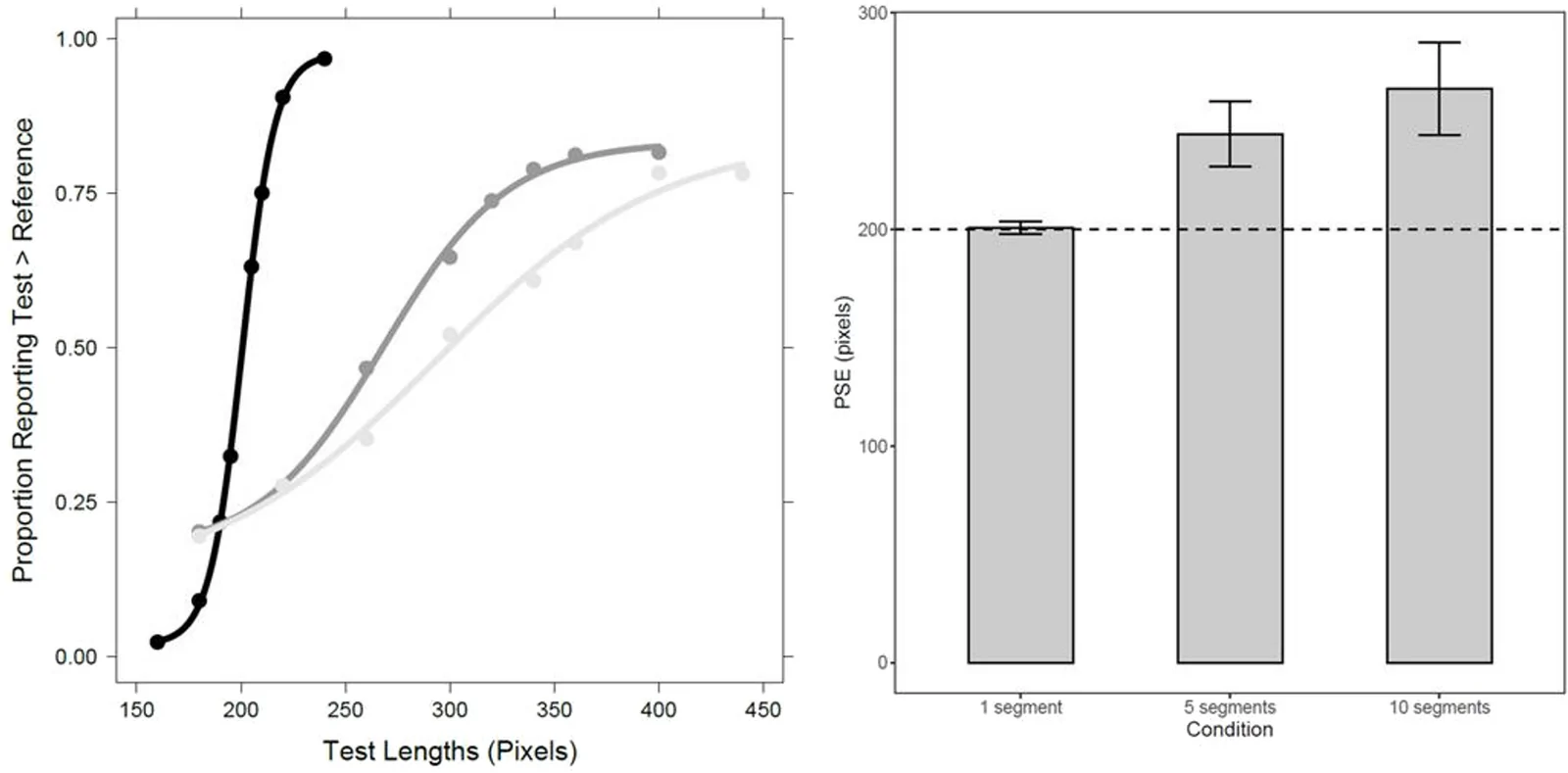
The psychometric functions averaged across participants for reporting the test to be greater than a reference is plotted for 1 (black), 5 (middle grey) and 10 (light grey) segment stimuli. The right hand panel plots the extracted mean PSEs and their associated 95% CIs

Figure 3 depicts the average psychometric functions obtained for comparing a reference with a single line (black), 5 segment (middle grey) and 10 segment (light grey) stimuli. The PSEs were, (*M* = 200.8, *CI* (197.8–203.8)) for single line; (*M* = 244.0, *CI* (228.8–259.1)) for 5 segments; and (*M* = 264.7, *CI* (243.4–286.1)) for 10 segments. Again, the PSE values for comparing a reference with a single line were similar to those in previous experiments. The PSEs were 22% & 32.35% for 5 & 10 segment conditions, and the underestimation of perceived size persisted.

To assess whether the absence of fixation point and the longer presentation duration modified the pattern of results, the data were analyzed using the same GLMM approach as in the previous experiments.

In the single line condition, the intercept and slope were both positive and significant (*Estimat*e = 3.09, *SE* = 0.15, *z* = 20.24, *p*< .001; *Estimat*e = 4.06, *SE* = 0.13, *z* = 31.39, *p*< .001) reflecting a steep psychometric function. Compared to the single line condition, intercepts were reduced for both 5 line segments (*Estimat*e = -3.0, *SE* = 0.08, *z* = -37.30, *p*< .001) and 10 line segments conditions (*Estimat*e = -3.30, *SE* = 0.08, *z* = -40.14, *p*< .001). This is consistent with the overestimation reflected on the PSE values since a greater physical length was required for the test stimulus to be perceived as equal in length to the reference stimulus. The interactions between stimulus length and line segments conditions were also significant (all *SE* ≈ 0.10, all *z* <-29 and all *p* < .001) for both 5 line segments (*Estimat*e = -3.00) and 10 line segments (*Estimat*e = -3.19). This indicates a shallower psychometric function and reduced discrimination precision.

In summary, the results of the two experiments do not support the notion that a reduction in perceived size with segmentation is caused by visual crowding.

## Experiment 3: Effect of statistical distribution

Apart from a perceptual bias, there are two other ways to explain the perceived size reduction with segmentation. These relate to a decisional bias rather than a perceptual bias. The first is that in all of the experiments above, in order to obtain a reliable estimate of the threshold and an accurate psychometric fit, there were more data points at longer test sizes. This meant that in effect there were more incidence of trials where the test stimuli were physically larger than the reference. Is it possible that as the experiment progressed, participants attempted to balance the number of times they chose reference and target as being larger? This possible approach would lead to choosing the reference a greater number of times, leading to the observed size underestimation of segmented lines. We have addressed this in experiment 3, where for both 5 and 10 segment tests, the incidence of physically longer and smaller tests were equal.

### Stimuli

For single line tests, the total stimulus length was 160, 180, 190, 195, 205, 210, 220 & 240 pixels. The total length for 5 and 10 segment stimuli was set from − 70% of the 200-pixel reference length to + 70% at 20% steps, that is 60, 100, 140, 180, 220, 260, 300 and 340 pixels.

### Procedures

Procedures were the same as those for experiment 1, in that after a fixation was shown for 500ms, the stimuli were presented for 250ms with a response window of 2000ms. There were 192 presentations (24 presentations at each of the 8 lengths) for each of the 3 experimental conditions. The analogy with pieces of gold when comparing the total length size was also removed and the participants were simply asked to report on the comparison of the total perceived length of stimuli.

### Participants

23 participants took part in this experiment (12 F, *M*_age_=36.61 *SD* = 12.00, *range* = 23–61).

## Results

**Fig. 4:**
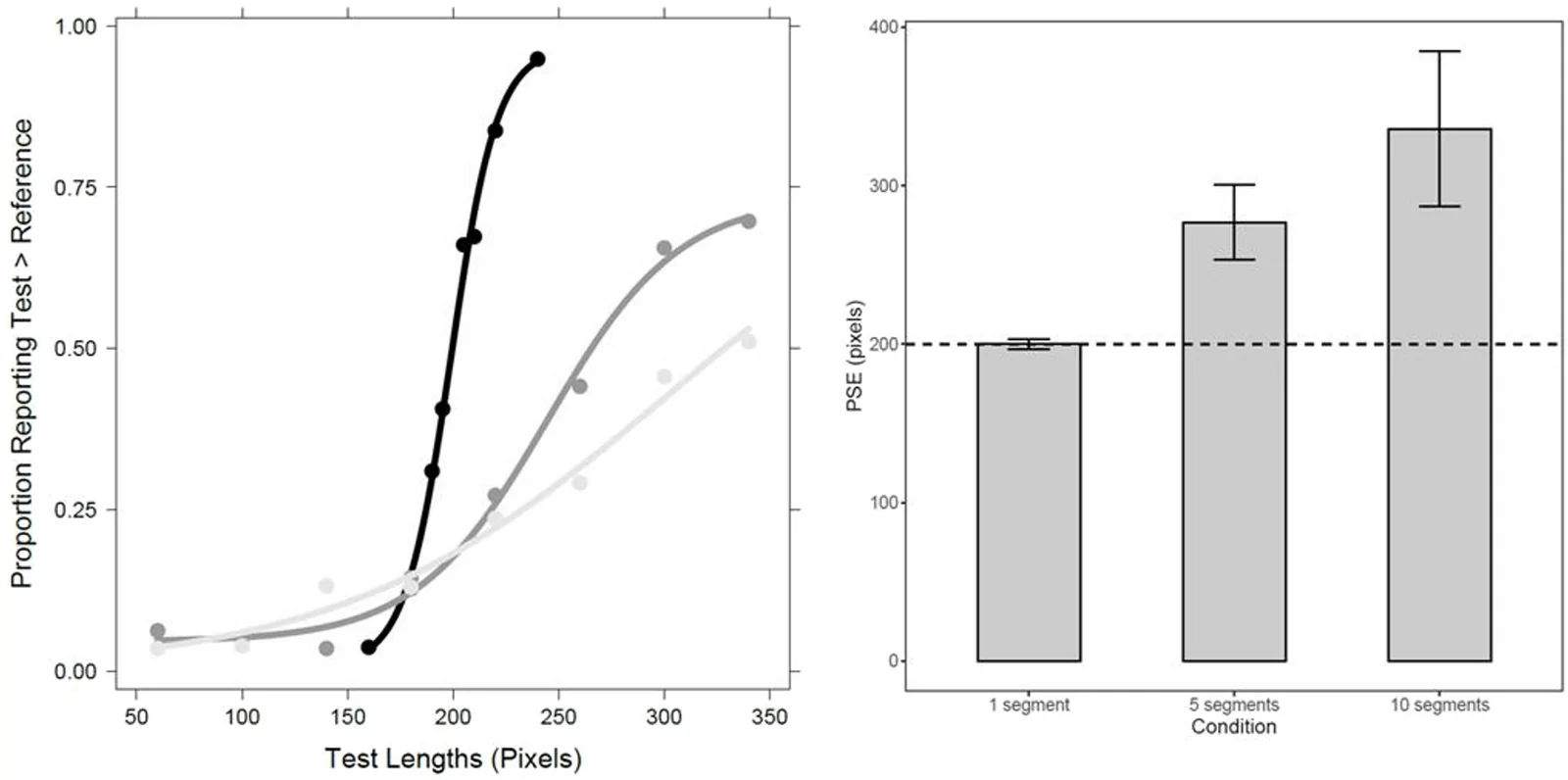
The psychometric functions averaged across participants for reporting the test to be greater than a reference is plotted for 1 (black), 5 (middle grey) and 10 (light grey) segment stimuli where the total number of trials where the test is greater than reference is equal to the number of trials where it is smaller. The extracted PSEs are shown on the right hand side. The error bars represent 95% CIs

Figure 4 depicts the average psychometric functions obtained for comparing a reference with a single line (black), 5 segment (middle grey) and 10 segment (light grey) stimuli. The PSEs (plotted on the right hand side) were, (*M* = 200.0, *CI* (196.7– 203.3)) for single line; (*M* = 276.8 (38.4%), *CI* (253.0–300.6)) for 5 segments; and (*M* = 335.6 (67.8%), *CI* (286.7–384.6)) for 10 segments. The findings demonstrate that the reported size underestimation is not caused by observers’ response based on statistical distribution of trials.

These results were confirmed by the GLMM. The intercept for the single line condition was indistinguishable from 0 (*Estimat*e < 0.001, *SE* = 0.008, *z* = 0.00, *p* = 1), indicating that the PSE closely matched to the 200 pixels reference length. This is consistent with the absence of any response bias in this condition. Moreover, the slope for length was large and positive (*Estimat*e = 3.781, *SE* = 0.083, *z* = 29.20, *p*< .001) indicating a steep psychometric function. Intercepts were significantly reduced, in comparison with the single line segment, for the 5 line segments (*Estimat*e = -0.844, *SE* = 0.036, *z* = -23.39, *p*< .001) and for the 10 line segments (*Estimat*e = -1.02, *SE* = 0.036, *z* = -28.55, *p*< .001). These shifts are consistent with the increase in PSE observed for segmented displays. The length × line segments interactions were also significant (*all SE* ≈ 0.006, all *z* < -27, and all *p*< .001) with more negative estimates in the 10 segments condition (*Estimat*e = -3.21) than in the 5 segments condition (*Estimat*e = -2.95). These negative interaction coefficients indicate shallower psychometric functions for segmented displays, indicating that discrimination precision decreased when line segments increased.

Supposing that participants, having encountered a difficult task of choosing between a single line or a harder task of perceptual manipulation, they made a decisional choice of reporting an intact line to be larger. This preference for choosing an intact line leads to an apparent size underestimation in the psychometric functions; hence a decisional bias may be a possible explanation for the findings. As the direction of behavioral effects from both decisional and perceptual bias are often the same, it remains a challenge for experimental designs to distinguish the two. Whether our findings are based on a true perceptual bias or if they are as a result of a decisional bias was addressed in experiment 4.

## Experiment 4: Effect of decisional bias

In a recently published study Linares and colleagues (Linares et al., 2019) have elegantly addressed this issue of distinguishing a decisional from a perceptual bias in a perceptual task of orientation discrimination. A homologue of their approach has been adapted to our task as follows. In a within-participant design, we compared the psychometric functions when participants reported which stimulus was larger, with the performance when they were required to respond which one was smaller. Then plotting the functions of the incidence of segmented lines being identified to be larger should lead to two overlapping functions for the two conditions if their response is based on a perceptual bias. On the other hand, a decisional bias (i.e., choosing an intact line for difficult judgments) would lead to two distinct psychometric functions appearing symmetrically with respect to the y-axis (see Fig. 1 of (Linares et al., 2019).

### Stimuli

1 and 5 segment stimuli were used in experiment 4 and they were of the same total length as in experiment 3.

### Procedures

For half the number of trials, participants reported the side of the screen that had a longer length and in the other half reported the shorter length side of the screen, the order of these was counterbalanced across participants. All other procedures, including instructions to participants were the same as experiment 3.

### Participants

19 participants (10 F, *M*_age_=34.84 *SD* = 11.92, *range* = 24–60) took part in experiment 4.

## Results

**Fig. 5:**
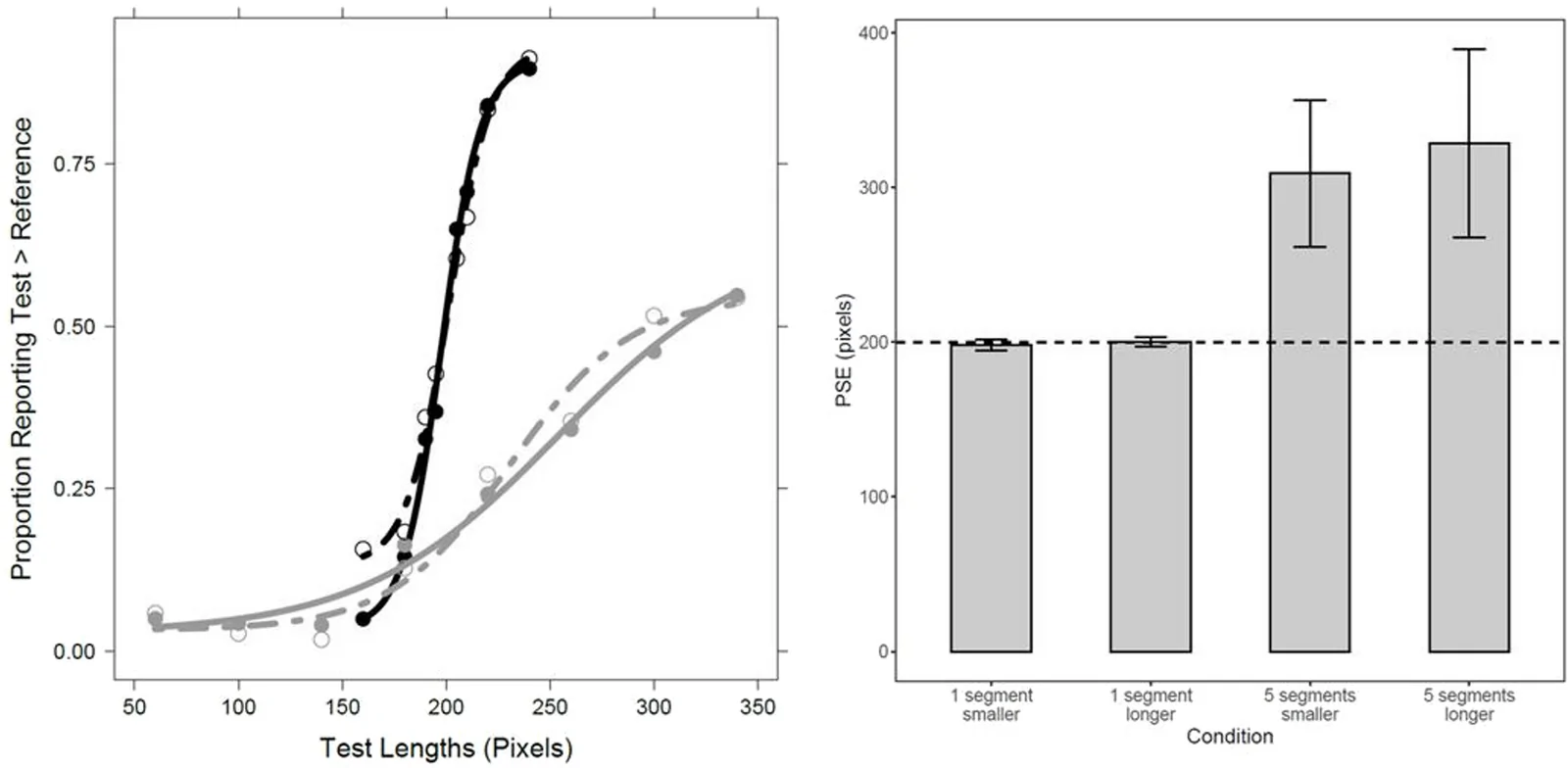
The psychometric functions for reporting which side had a longer length (solid lines) and for reporting which side had a shorter length (broken lines) are plotted for 1 (black) and 5 (middle grey) segment conditions on the left panel. The corresponding extracted PSEs from the fitted probit functions are shown on the right hand panel. Error bars represent 95% CIs

Figure 5 shows data for single and 5-segment stimuli under two reporting conditions. Participants reported which side was longer (solid lines) or smaller (dotted lines). Data represents the incidence of a test appearing longer than reference. For solid lines, the PSEs for reporting shorter or longer stimuli of single lines were (*M*_*longer*_=200.1, *CI* (197.2–203.01)) and (*M*_*shorter*_=198.0, *CI* (194.4– 201.05)); and (*M*_*longer*_=328.5 (64.25%), *CI* (267.7– 389.2)) and (*M*_*shorter*_=309.0 (54.5%), *CI* (261.6– 356.4)) for 5 segment stimuli respectively.

The analysis procedure was identical to that used in Experiment 1. In the reference condition (single line segment, larger task), the intercept was not significant (*Estimat*e = -0.006, *SE* = 0.067, *z* = -0.10, *p* = .924), and the slope for length was significantly positive (*Estimat*e = 2.93, *SE* = 0.14, *z* = 20.88, *p*< .001), indicating a steep psychometric function and high discrimination precision. In the single line segment condition, the “smaller” judgment task differed slightly from the “larger” judgment task, the intercept was small but significant (*Estimat*e = 0.079, *SE* = 0.034, *z* = 2.32, *p*< .02). In contrast, both 5 line segments conditions showed a significantly reduced intercept in comparison to the single line segment (all *SE* ≈ 0.039, all *z* < -24, all *p*< .001) with an estimate of -1.03 for the “larger” judgment task and an estimate of -0.98 for the “smaller” judgment task. These shifts are consistent with the increase in PSE observed for segmented displays, indicating that greater physical lengths were required for the stimuli to be perceived as equal in length to the reference stimulus. The Interaction between stimulus length and the two 5 line segments conditions were also significant (all *SE* ≈ 0.094, all *z* < -24, all *p*< .001), indicating shallower psychometric functions and a reduced discrimination precision relative to the reference stimulus.

The similarity of the psychometric functions under the two reporting conditions and the overlapping confidence intervals of the extracted PSEs demonstrate that decisional bias cannot explain the observed reduction in perceived size with segmentation and the reported observations are more likely to be as a result of a perceptual bias. We aimed to extend the computer based task to laboratory using real objects as a step to explore the findings in the context of observations in daily life. This was addressed in the next experiment.

## Experiment 5: Expansion to real objects under laboratory settings

**Fig. 6:**
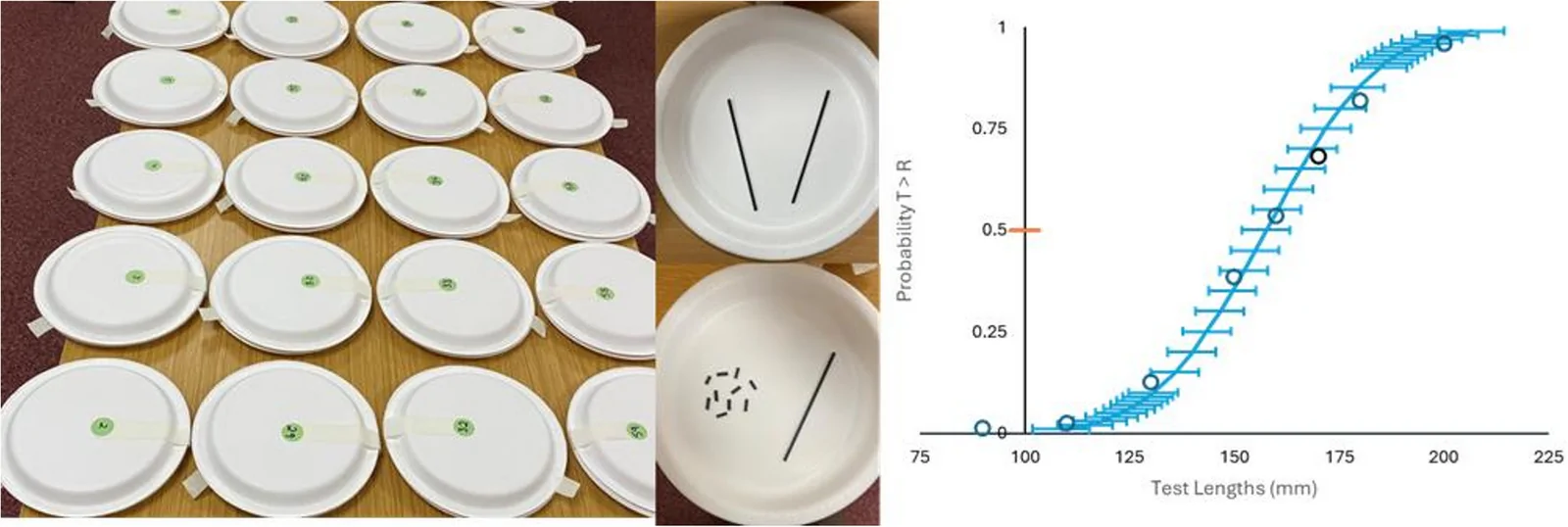
The experimental setup and example stimuli for physical objects presented under laboratory conditions are shown on the left panel. The psychometric function for discrimination of total length for 10 segment stimuli against a 100 mm reference line, averaged across all participants is shown on the right panel (95% CI for fitted function indicated). The discrimination threshold range for a single line test is indicated by a red line

We aimed to extend the ecological validity of our findings by using real objects under laboratory conditions. To do this, we needed to set up repeated observations of real objects, recording reported verbal responses of participants. Figure 6 shows a set up where precision-cut pieces of cardboard stimuli consisting of lines and segments were placed on paper plates.

### Stimuli

Stimuli were constructed using thin black cardboard. The cardboard chosen was a black sheet of 80 × 120 cm 1.3 mm thickness (Ref 663600300 La Cite des Arts, Rennes, FR) precision cut into 2 mm strips. The reference stimuli were 100 mm stripes, accurately measured to 0.1 mm. The 8 test lengths used were 90, 110, 130, 150, 160, 170, 180 & 200 mm in length. These were then divided into 10 segments of approximately equal length. Stimuli were placed on 17 cm white plates as shown in Fig. 6 with 12 stimuli per length, totaling 96 plates. The left and right order of reference/test were counterbalanced, and the order of all plates were based on a random order sequence. A transparent circular overlay of 5 cm diameter was used to limit the extent of the distribution of segments on the plates. There were 4 training plates to demonstrate the task and were shown prior to the experiment.

### Procedures

Participants were shown one plate at a time and asked to report the side of the plate that contained more length. Plates 1–96 were shown sequentially, followed by a reverse-order presentation of plates 88–1, followed by plates 96–89, providing 192 observations per participant. In each trial, one plate at a time was momentarily uncovered by the investigator. Participants verbally reported the side of the plate (left/right) that they perceived to have a greater length, using the same analogy of gold bars as reported in experiment 1.

Two training plates were used to demonstrate size discrimination for the two solid lines. These contained comparisons of a 100 mm reference with a 90 mm and 110 mm test lines respectively. The method of limits was used to establish the discrimination threshold by estimating the lower and upper thresholds for detecting a difference between the lengths of a reference and a test. To obtain the lower threshold, plates containing a reference and a test starting at 95 mm were shown. Five plates in which the test length was incremented in 1 mm steps (95–100 mm), were shown sequentially. The task was to correctly identify the test (which one is shorter? ), or if they appear to be the same length. The previous plate to the one wrongly identified or reported to be the same was noted as the lower threshold. The task was repeated, starting with a 105 mm test to determine the upper threshold where participants reported which line was longer. Photographs of the experimental setup and all the plates used can be found on the Open Science Forum (https://osf.io/xubpm/).

### Participants

24 participants (12 F, *M*_age_ = 30.63, *SD* = 13.62, *range* = 20–62) took part in experiment 5. They were recruited on the campus of the University of Aberdeen and were not paid.

## Results

The graph in Fig. 6 plots the average psychometric function fitted to the data of 24 participants, together with the 95% confidence interval of the fitted curve, when observers reported which of the reference (100 mm) or the test stimuli (10 segments) were longer. In the same participants, using the method of limits, the threshold for PSEs for comparison of two lines were in a range of (± 3.4%) (*CI* (96.54–103.42) and were comparable to classical observations. The average PSE for 10 segment stimuli was (*M* = 157.93 (57.93%), *CI* (156.63–159.07)) indicating that a 10-segment real object would need to be 57.9% longer (in length) to be perceived as equal to an intact standard line.

The findings demonstrate that a significant reduction in perceived size of segmented lines is maintained for real objects under laboratory conditions. If the findings could be generalized to other real-world daily interactions, this would have significant implications on many activities of daily living.

## Experiment 6: Behavioral interactions with food stimuli

In two further investigations outlined below, we have extended our observations to behavioral interactions with food stimuli resembling line segments. All the findings reported so far in this manuscript utilized a forced-choice discrimination methodology to obtain psychometric functions relating perception of size to its physical dimensions. Here we have also expanded in our methodological approach. We presented the observers with a single strand of spaghetti of fixed size and using a matching task we asked participants to match the observed quantity using spaghetti pieces of varying length (experiment 6 A). In a separate study, we simply asked participants to place enough spaghetti on a plate, equivalent to a portion size (6B), removing the comparison with a reference object.

### Stimuli

Spaghetti strands (Tesco UK, Organic Spaghetti, 539 kJ 127 kcal per 100 g) were par-cooked (2 min), drained, and covered in vegetable oil to maintain texture. They were cut into 1, 2, 5, 10 and 20 cm pieces using a metal ruler, and placed in separate clear food boxes for storage. Standard white plates (23 cm diameter) were placed in front of participants.

### Procedures

Experiment 6 A involved a length matching task where participants observed a 20 cm piece of cooked spaghetti (standard) placed diagonally on a white plate. Participants were asked to place in the center of an empty white plate, from a box of cut spaghetti, the amount that matched the length of the standard. The boxes containing cooked spaghetti, cut into 1, 2, 5 and 10 cm pieces were presented with order counterbalanced across participants. The total number of pieces of spaghetti for each condition was recorded. The final experiment (6B) was to examine the reduction in perceived length of segmented stimuli to a real-life condition without a physical reference. We asked the participant to place on a plate the amount of spaghetti that they perceived as being equivalent to one portion size, again the order of cut pieces of spaghetti were randomized.

### Participants

28 participants (17 F, *M*_age_ = 30.50, *SD* = 11.22, *range* = 19–57) took part in both experiments 6 A and 6B, the order of which was counterbalanced between participants. Participants were recruited via opportunity sampling and were paid £10 for their participation.

## Results

**Fig. 7:**
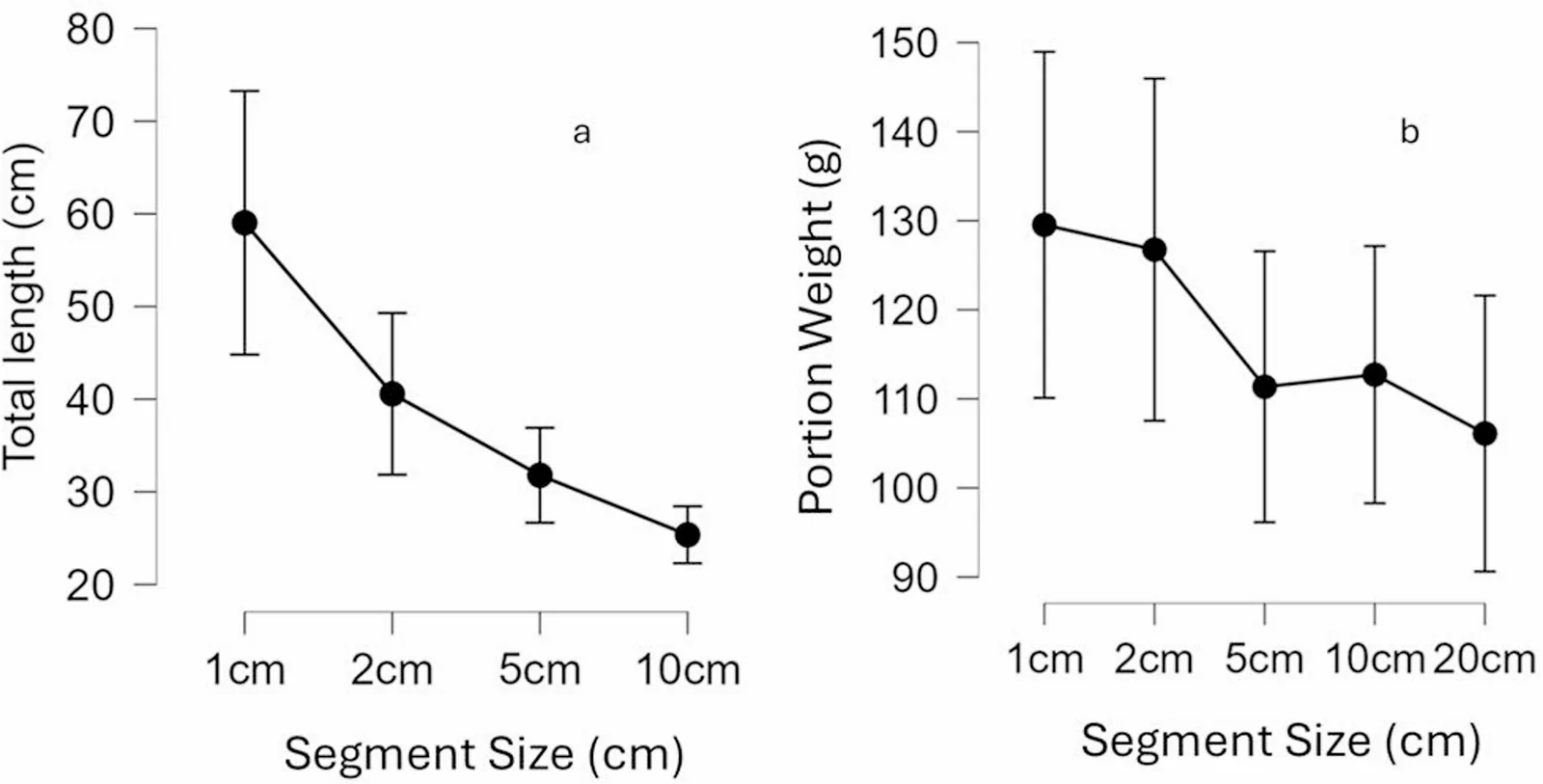
The total length of spaghetti pieces to match a 20 cm reference spaghetti strand plotted as a function of segment length, averaged across participants is shown in panel (**a**). On the right panel (**b**) shows the average weight of spaghetti considered to be one serving portion, as a function of the segment length, averaged across participants

The average length of spaghetti matched with the reference for all the participants is plotted in Fig. 7a. Analysis of variance showed a significant main effect of segment size in the matching length task (F(1.81, 48.94) = 17.58 Greenhouse-Geisser corrected, *p* < .001 η_p_^2^ = 0.394). Participants underestimated the reference length by 26.8%, 58.9%, 102.9% and 195.2% for spaghetti sizes of 10 cm, 5 cm, 2 cm and 1 cm respectively, with differences between 1 cm and other conditions; 2&10; and 5&10 cm all being statistically significant (all p_bonf_ <0.05).

If in a real-life task the size underestimation of items consisting of small segments persisted, one would expect that larger weights of spaghetti are placed on a plate with reduced segment size. Figure 7b shows that this was indeed the case. Analysis of variance showed that there was a main effect of segment size (F(2.61, 70.57) = 7.83 Greenhouse-Geisser corrected, *p* < .001 η_p_^2^ = 0.225), with participants on average placing 6.6%, 5.3%, 20.6% and 23.4% more food (in grammes of weight) on a plate for 10, 5, 2 & 1 cm cut spaghetti compared to 20 cm cut spaghetti. The differences between 1 cm with 5 & 20, as well as 2 and 20 cm being significant (all p_bonf_ <0.05).

Observations in experiment 6 A extend the findings from forced-choice size discrimination task to a real-life size-matching task, demonstrating that the participants underestimated perceived size in a matching task depending on the segment length. The findings from 6B show the same pattern of results, when the physical reference has been removed. In portion estimation task, in addition to segment size, there are several other factors that can contribute to the size estimation such as the perceived volume, distribution of the pieces and occlusion by overlapping segments which in turn could provide further clues to portion estimation. Nevertheless, despite the additional cues, the perceived size reduction with segmentation remains.

## Discussion

We have provided psychophysical and behavioral evidence for a systematic underestimation of perceived length judgement for segmented line stimuli. This phenomenon persists, irrespective of viewing time and extends also to real objects under laboratory conditions. Remarkably, it can also be observed in a real object matching task and its effect can be observed in an everyday task by influencing the amount of food the participants expect to be equivalent to a portion size. In the context of magnitude estimation based on sensory comparisons, the perceptual size underestimation with segmentation reported here is large, notably, if a line object is broken into 10 pieces, the observers perceive their collective size to be 60% of the length of the intact line. This is much larger than the just noticeable differences between nearly identical line stimuli which are in the order of 1–3%.

The neuronal coding for line stimuli relies on spatial integration along its contour. The evidence for spatial integration of stimulus intensity (and size) was reported with the discovery of receptive fields for a single cells (Hartline, 1938). The spatial integration of cells enabling coding of the contours for continuous bars/lines was reported at the dawn of the modern visual neurobiology (Hubel & Wiesel, 1959). The subsequent psychophysical investigations of perceptual abilities in human observers demonstrated the high sensitivity for spatial integration leading to detection of faint contours in the form of grating patterns (Campbell & Robson, 1968), with the measurement of their detection thresholds resulting in the discovery of Contrast Sensitivity Function. More recently, detailed psychophysical investigations using suprathreshold elongated (line-like) stimuli of small and large sizes (10’-300’ arc) have been conducted to reveal the spatial summation (magnitude coding) properties for mechanisms encoding stimulus length (Chen et al., 2019, 2023). They revealed the existence of psychophysical channels coding lengths ranging from small targets to a long-range channel extending an entire hemifield (Chen et al., 2023). These findings are in agreement with the neurophysiological studies showing coding of length in cats visual cortex (DeAngelis et al., 1994; Gilbert & Wiesel, 1989).

A large body of research spanning from single cell recording to psychophysical investigations on detection of contours, the spatial summation and integration along their length to allow coding of their extent indicate precision in determination of line-object extent. The discrimination thresholds of 1–3% for judgment of a single line are therefore not surprising. For multiple objects, although the raised variance is predictable due to associated internal noise, the consistent findings of elevated thresholds for accumulated length are surprising. Size perception is a complex psychological phenomenon where the initial coding of the objects’ physical properties is interpreted based on psychological priors. So, the size of a given physical dimension can be perceived as being different depending on the context and the application of priors (Todorović, 2010) leading to size illusions, such as müller-Lyer (Du Bois-Reymond, 1889), Ebbinghaus (Titchener, 1899) and Ponzo illusions (Bertamini & Wade, 2023) to name a few. Here we have provided evidence in support of the presence of a psychological prior where the magnitude of length for segmented real line objects is perceived to be lower than an intact line stimulus. The classical, well-known Oppel-Kundt illusion refers to the perceived length of imaginary lines that are represented by their endpoint (Luckiesh, 1922). When the space between two end points forming an imaginary line is segmented by multiple physical objects, the imaginary line is perceived to be longer than another imaginary line without such subdivisions (Kundt, 1863), although curiously a single division (or bisected line) lead to an opposite perception of reduction of length (Mamassian & de Montalembert, 2010; Mikellidou & Thompson, 2014). In the spatial coding account of this illusion, it is hypothesized that the presence of segments leads to higher neuronal activation (Bulatov et al., 2020) signaling a longer length. The findings reported here across 8 experiments, using both psychophysical and behavioral tasks, consistently show an effect in the opposite direction for real objects where higher number of discrete items leads to a lower perceived length. Conflicting accounts in classical illusions have previously been documented (Deręgowski, 2015) which together with observations reported here perhaps demonstrate the wide range of priors and their interactions that lead to size perception.

In adults, size differences for nearly identical line stimuli can be detected with high precision as reported in the classical psychophysical investigations (Fechner, 1860; Weber, 1996). For multiple objects stimuli, ensembled perception refers to experimental conditions where summary statistics are extracted from a large number of test objects. For example, when participants report the average brightness of a large set of targets, each at varying brightness levels (Bauer, 2009). Indeed high precision of ensembled perception of basic stimulus attributes such as orientation, color, and position as well as higher level information such as facial emotions or identities even at short presentations (200ms) have been reported (Neumann et al., 2018; Yamanashi Leib et al., 2014), see (Whitney & Leib, 2018) for review). The accuracy is also high for ensembled perception of average line size (Miller & Sheldon, 1969) or width of circular patches (Ariely, 2001; Kacin et al., 2021; Yildirim et al., 2018 Yörük & Boduroglu, 2020). Extracting summary statistics in relation to size has high accuracy and may rely on a sampled small subset of the stimulus set (de Fockert & Marchant, 2008; Solomon et al., 2011). Due to high correlation between some summary statistics, not all parameters have to be determined and it is indeed not efficient to do so, and some can be derived from the others such as numerosity and average size (Lee et al., 2016). We postulate that perception of aggregate magnitude, or in this case total length estimation, is not finely tuned and has priors that lead to systematic underestimation. However, an alternative explanation of the underlying mechanism leading to the perceived length reduction may relate to errors in estimation of numerosity and average size. Combining numerosity and ensembled average size has been shown effective in explaining the accumulated perceived surface area (Lee et al., 2016). It remains to be tested whether a similar approach could explain the present findings. A further alternative explanation for the findings may relate to precision of coding of the segment endpoints. If the coding is not accurate and it leads to a segment being perceived as smaller in size, then further segmentation would lead to more errors and elevated PSEs. This possibility can be explored by investigating the accumulated length when stimuli are segmented *by qualitative properties (such as distinct textures or colors) rather than by physical spatial separation.*

Overall, the findings reported here are contrary to expectations. The paradox is the presence of a systematic error for perception of cumulative length, where all psychophysical and psychophysiological evidence points to precision for coding of individual elements. As far as the underlying mechanisms for the findings are concerned, from a putative Bayesian perspective, there are two sources of variance. One is related to the sensory coding (posterior probability) whose noise is augmented with increasing number of elements to be integrated. A second source of noise would probably relate to a top/down prior probability, predicting diminished magnitude with segmentation. In this model, the former source of variance, leads to a shallower slope of the psychometric functions with increasing segmentation, whereas the latter determines the level of perceptual bias (rightward shift). In this configuration, the prediction of the model would be that if the single line reference is replaced with multiple segments at a fixed cumulative length, then the psychometric functions (at different segmentations) would pivot at the center (no rightward shift) due to the constant prior. The variation in the just noticeable difference (JNDs) of such psychometric functions at different levels of segmentations would solely relate to sensory encoding, being smaller than those reported for single line reference. These predictions will be investigated in future experiments.

For the experiments reported here, PSEs for total length judgements changed monotonically with increasing number of line segments. However, the determined thresholds for a given number of segments were not always consistent across the conditions. For example, thresholds PSEs for 5 segment stimuli were 39.1% in experiment 1, but interleaving stimulus presentation times led to threshold of 72.5% and 66.3% (experiment 2 A) but were reduced to 22% for uniformly long (experiment 2B) or short presentation durations (38.4% in experiment 3 A). Nevertheless, they were different than those in experiment 3B (64.25 and 54.5%). Considering the individual participant data in these cohorts (plotted in supplementary material), it appears that some participants would consistently report segmented lines to be shorter than reference for most line magnitudes, i.e., segmentation had a large effect on their performance. It is likely that whilst there is consistency in relative relationships between conditions in each experiment, the absolute values measured may differ across experiments, depending on the number of such participants in each random cohort.

The current theories for extracting magnitude for surfaces and volumes rely on multiple estimates of dimensions by observers (Yousif & Keil, 2020), the findings reported here have significant implications for developing theories of magnitude perception and may point to sources of divergence between predicted and perceived magnitudes (Morgan, 2005; Nachmias, 2011; Yousif & Keil, 2021).

The findings also have practical implications, for example in the field of nutrition. The perception of the food quantity has been shown to be affected by subjective food preferences (Bar et al., 2025). Here extending the experimental findings to everyday activities, we have demonstrated the persistence of a systematic length underestimation using food stimuli. This effect, when applied to food portions, was significant (22% more quantity for smallest pieces, compared to the longest length) which in effect, can lead to a proportional increase in calorie intake. The interactions between food shape and size perception are certainly worth further investigation in view of these observations.

In summary, we have demonstrated a perceptual bias in estimating the length of segmented lines in that they are observed to be smaller in aggregate size, compared to intact lines. The findings extend to real objects and even to an example of food quantity. Understanding the limits of these biases and factors affecting them would be of great interest.

## Supplementary Information


Supplementary Material 1.


## Data Availability

Prior to conduct of this study, details of studies to be conducted, expected outcomes, inclusion/exclusion criterion as well as planned analysis were placed on Open Science Forum. These, as well as all data, codes and protocol documents and notes can be accessed on Open Science Forum (https://osf.io/xubpm/).
