# Supplementary material for: The length of a piece of string: Where the whole is more than the sum of its constituent parts
Source: Psychol Res. 2026 Jun 29;90(4):121. doi: 10.1007/s00426-026-02336-z (PMC13315493; doi:10.1007/s00426-026-02336-z)
Supplement: Supplementary file 1 — Supplementary Material 1. [file 426_2026_2336_MOESM1_ESM.docx]

**Supplementary material**

**The length of a piece of string: where the whole is more than the sum of its constituent parts**

Frédéric Devinck^1^, Olivier Le Bohec^1^, Alexandra M Johnstone^2^, Arash Sahraie^1,3^*

^1^Département de psychologie, Université Rennes 2, Rennes 35000, France

^2^Rowett Institute, School of Medicine, Medical Sciences and Nutrition, University of Aberdeen, Aberdeen AB24 3FX, UK

^3^School of Psychology, University of Aberdeen, Aberdeen AB24 3FX, UK

Correspondence concerning this article should be addressed to Arash Sahraie, Department of Psychology, William Guild Building, University of Aberdeen, Aberdeen AB24 3FX, United Kingdom Email: [a.sahraie@abdn.ac.uk](mailto:a.sahraie@abdn.ac.uk)

**Pilot investigation**

Prior to the conduct of experiments reported in this manuscript, a pilot investigation was performed to establish the appropriate range of parameters, experiment durations, size of the effect, appropriate sample size, appropriateness of inclusion, exclusion criterion, and the efficacy of online testing for these investigations. All the rationale and description of the studies were pre-registered and the time stamped documents can be accessed on Open Science Forum (https://osf.io/xubpm/).

Data on 24 observers were recorded using Testable Minds in order to determine psychometric functions for experimental conditions of 1, 2, 3, 4 & 5 line segments. A schematic diagram of sequence of fixation, stimuli and response for comparing two lines, or a reference with 2 and 5 line segments are shown below.


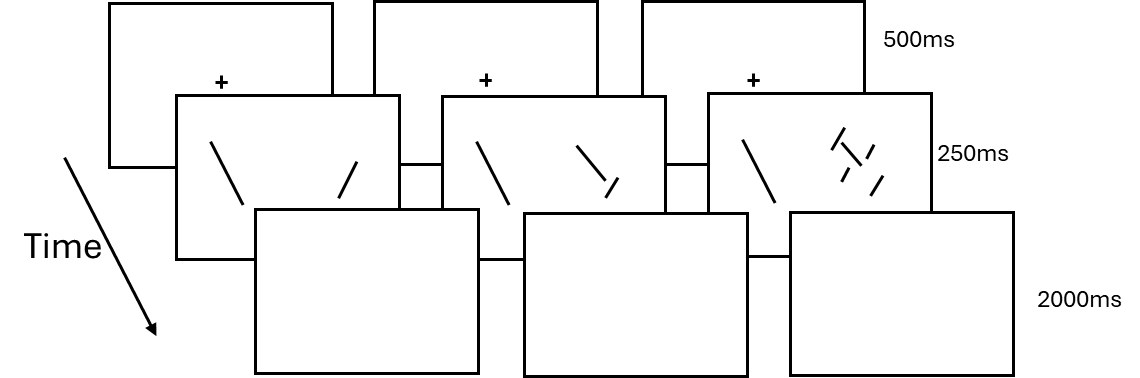


A priori, we had defined an invalid trial as a trial where the participant did not respond (RT>2000ms) or those with rapid response (RT <200ms). Those with more than 8% invalid trials were excluded. Participants could also miss catch trials. This is important as the catch trials were to determine if the participants have paid attention to the task. If 6 or more out of 24 catch trials were responded to incorrectly, then the participant was excluded. This led to exclusion of 4 participants. However, the data of P11 and P18 also shows that there were participants that responded in a random manner to the task in the simplest form, i.e., comparing size discrimination for a single line against a reference. Although for majority of participants this leads to a sharp psychometric function, this was not the case for these two participants indicating that they could not perform the task. For this reason, the exclusion criterion was revised (version 2.0 on pre-registration) to state that a participant is excluded in the absence of a defined psychometric function for comparison of two lines, with the rationale that if they cannot reliably make comparison judgements between two lines, they would be incapable of reliably reporting size differences for multi-segment stimuli.

Another observation on the pilot study is that for fitting a psychometric curve, a wider range of length sizes would need to have been included, that would lead to a better curve fitting and higher confidence on the extraction of the point of subjective equality. The final observation on the pilot data is that the psychometric curves become shallower and seem to merge for 4 and 5 segment conditions. Therefore, there is a need to include a condition with larger number of segments (i.e., 10 segments) to determine if there is a limit of 4 segments for this task. In addition, for the subsequent experiment, we increased that number of recruited participants to 34 to ensure a minimum of 20 data sets would satisfying our inclusion criterion.

Data for all participants in the preliminary experiment are shown below.

**Fig.1** Individual participant data for pilot investigation. The probability of reporting the test to be longer as a function of test size for 1 (black), 2 (dark grey), 3 (middle grey), 4 (light grey) and 5 (off white) segment stimuli are plotted


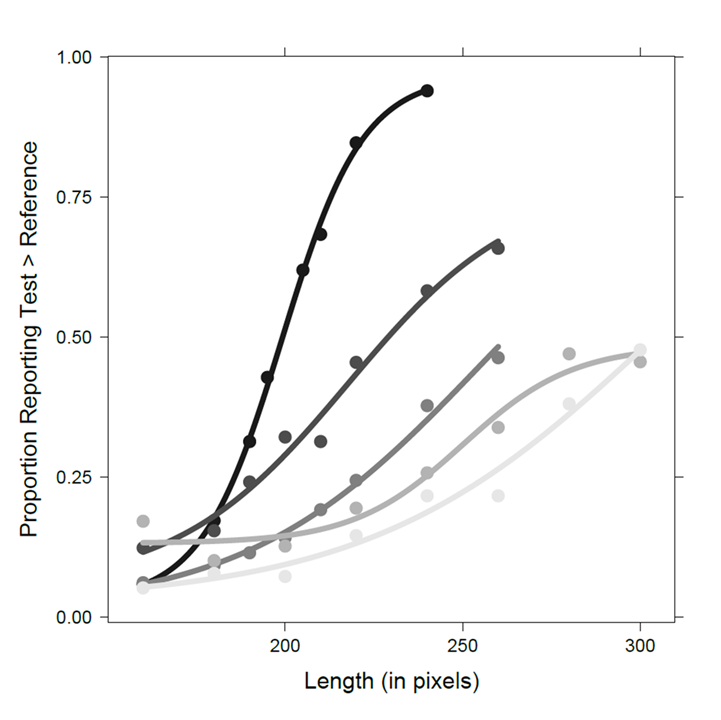


**Fig 2** the average psychometric functions for reporting the test to be longer than the reference line or 1 (black), 2 (dark grey), 3 (middle grey), 4 (light grey) and 5 (off white) segment stimuli

**Fig 3** PSEs are plotted on the left panel and the JNDs are displayed on the right panel. Error bars indicate 95% CIs

**Table 1.** PSE and JND estimates (in pixels) with 95% confidence intervals derived from the delta method.

| **Condition** | **PSE** | **95% CI** | **JND** | **95% CI** |
| --- | --- | --- | --- | --- |
| 1 segment | 199.6 | [192.8, 206.3] | 16.2 | [14.1, 18.3] |
| 2 segments | 229.7 | [210.3, 249.1] | 36.1 | [26.1, 46.0] |
| 3 segments | 269.2 | [235.5, 302.9] | 43.5 | [28.9, 58.2] |
| 4 segments | 334.1 | [225.6, 442.6] | 90.3 | [29.4, 151.2] |
| 5 segments | 332.6 | [255.5, 409.8] | 64.7 | [33.2, 96.2] |

**Table 2.** Fixed effects of the GLMM (probit link). The reference condition is 1 segment.

| **Parameter** | **Estimate** | **SE** | **z** | **p** |
| --- | --- | --- | --- | --- |
| Intercept | 0.644 | 0.163 | 3.96 | < .001 |
| Length | 1.601 | 0.107 | 14.94 | < .001 |
| Condition: 2 segments | −0.920 | 0.039 | −23.72 | < .001 |
| Condition: 3 segments | −1.483 | 0.041 | −36.54 | < .001 |
| Condition: 4 segments | −1.534 | 0.042 | −36.82 | < .001 |
| Condition: 5 segments | −1.871 | 0.045 | −41.47 | < .001 |
| Length × 2 segments | −0.883 | 0.058 | −15.28 | < .001 |
| Length × 3 segments | −1.006 | 0.060 | −16.89 | < .001 |
| Length × 4 segments | −1.314 | 0.053 | −24.59 | < .001 |
| Length × 5 segments | −1.200 | 0.055 | −21.94 | < .001 |

**Experiment 1**

Data for all participants in Exp 1 is shown below.

**Fig. 4** Individual participant data for Exp 1. The incidence of reporting the test to be greater than a reference for the 4 experimental conditions are plotted for 1 (black), 2 (dark grey), 5 (middle grey) and 10 (light grey) line segments.

**Fig 5** Results of JNDs for Exp1 . Error bars indicate 95% CIs

**Table 3.** Fixed effects of the GLMM (probit link). The reference condition is 1 segment.

| **Parameter** | b | **SE** | z | p |
| --- | --- | --- | --- | --- |
| Intercept | 3.539 | 0.165 | 21.39 | < .001 |
| Length | 4.085 | 0.148 | 27.65 | < .001 |
| Condition: 2 segments | −2.261 | 0.111 | −20.30 | < .001 |
| Condition: 5 segments | −3.726 | 0.110 | −34.02 | < .001 |
| Condition: 10 segments | −4.162 | 0.110 | −37.91 | < .001 |
| Length × 2 segments | −2.437 | 0.129 | −18.95 | < .001 |
| Length × 5 segments | −3.035 | 0.123 | −24.77 | < .001 |
| Length × 10 segments | −3.550 | 0.121 | −29.39 | < .001 |

**Experiment 2A & 2B**

Data for all participants in Exp 2A is shown below.

**Fig.6** The individual participant data for reporting the test to be greater than a reference is plotted for 1 (black), 3 (dark grey) and 5 (light grey) segment stimuli at short (solid lines) and long (broken lines) randomly interleaved presentation times, as a function of test length.

**Fig 7** Results of JNDs for Exp2A. Error bars indicate 95% CIs

**Table 4.** Fixed effects of the GLMM (probit link). The reference condition is 1 segment.

| **Parameter** | **Estimate** | **SE** | **z** | **p** |
| --- | --- | --- | --- | --- |
| Intercept | 2.953 | 0.167 | 17.73 | < .001 |
| Length | 3.206 | 0.127 | 25.23 | < .001 |
| Condition: 3 segments (1000 ms) | −2.893 | 0.092 | −31.52 | < .001 |
| Condition: 3 segments (250 ms) | −2.999 | 0.092 | −32.73 | < .001 |
| Condition: 5 segments (1000 ms) | −3.704 | 0.094 | −39.42 | < .001 |
| Condition: 5 segments (250 ms) | −3.726 | 0.094 | −39.71 | < .001 |
| Length × 3 segments (1000 ms) | −1.960 | 0.100 | −19.54 | < .001 |
| Length × 3 segments (250 ms) | −2.151 | 0.100 | −21.61 | < .001 |
| Length × 5 segments (1000 ms) | −2.547 | 0.097 | −26.36 | < .001 |
| Length × 5 segments (250 ms) | −2.622 | 0.096 | −27.19 | < .001 |

All participant data for Exp 2B is shown below.

**Fig. 8** The individual participant data for reporting the test to be greater than a reference is plotted for 1 (black), 5 (middle grey) and 10 (light grey) segment stimuli.

**Fig 9** Results of JNDs for Exp2A. Error bars indicate 95% CIs

**Table 5.** Fixed effects of the GLMM (probit link). The reference condition is 1 segment.

| **Parameter** | **Estimate** | **SE** | **z** | **p** |
| --- | --- | --- | --- | --- |
| Intercept | 3.086 | 0.152 | 20.24 | < .001 |
| Length | 4.058 | 0.129 | 31.39 | < .001 |
| Condition: 5 segments | −2.999 | 0.080 | −37.30 | < .001 |
| Condition: 10 segments | −3.299 | 0.082 | −40.14 | < .001 |
| Length × 5 segments | −3.002 | 0.102 | −29.47 | < .001 |
| Length × 10 segments | −3.186 | 0.100 | −31.84 | < .001 |

**Experiment 3**

All participant data for Exp 3 are plotted below.

**Fig. 10** Individual participant data for reporting the test to be greater than a reference is plotted for 1 (black), 5 (middle grey) and 10 (light grey) segment stimuli where the total number of trials where the test is greater than reference is equal to the number of trials where it is smaller

**Fig 11** Results of JNDs for Exp2A. Error bars indicate 95% CIs

**Table 6.** Fixed effects of the GLMM (probit link). The reference condition is 1 segment.

| **Parameter** | **Estimate** | **SE** | **z** | **p** |
| --- | --- | --- | --- | --- |
| Intercept | < 0.001 | 0.083 | 0.00 | 1.000 |
| Length | 3.781 | 0.130 | 29.20 | < .001 |
| Condition: 5 segments | −0.844 | 0.036 | −23.39 | < .001 |
| Condition: 10 segments | −1.018 | 0.036 | −28.55 | < .001 |
| Length × 5 segments | −2.945 | 0.106 | −27.75 | < .001 |
| Length × 10 segments | −3.210 | 0.106 | −30.32 | < .001 |

**Experiment 4**

Individual participant data for Exp 4 are plotted below.

**Fig. 12** Individual participant data for reporting which side had a longer length (solid lines) and for reporting which side had a shorter length (broken lines) are plotted for 1 (black) and 5 (middle grey) segment conditions

**Fig 13** Results of JNDs for Exp2A. Error bars indicate 95% CIs

**Table 7.** Fixed effects of the GLMM (probit link). The reference condition is 1 segment (Larger).

| **Parameter** | **Estimate** | **SE** | **z** | **p** |
| --- | --- | --- | --- | --- |
| Intercept (1 segment, Larger) | −0.006 | 0.067 | −0.10 | .924 |
| Length | 2.927 | 0.140 | 20.88 | < .001 |
| Condition: 1 segment (Smaller) | 0.079 | 0.034 | 2.32 | .020 |
| Condition: 5 segments (Larger) | −1.030 | 0.039 | −26.30 | < .001 |
| Condition: 5 segments (Smaller) | −0.982 | 0.039 | −24.93 | < .001 |
| Length × 1 segment (Smaller) | −0.527 | 0.121 | −4.36 | < .001 |
| Length × 5 segments (Larger) | −2.388 | 0.094 | −25.46 | < .001 |
| Length × 5 segments (Smaller) | −2.321 | 0.094 | −24.73 | < .001 |

**Experiment 5**

**
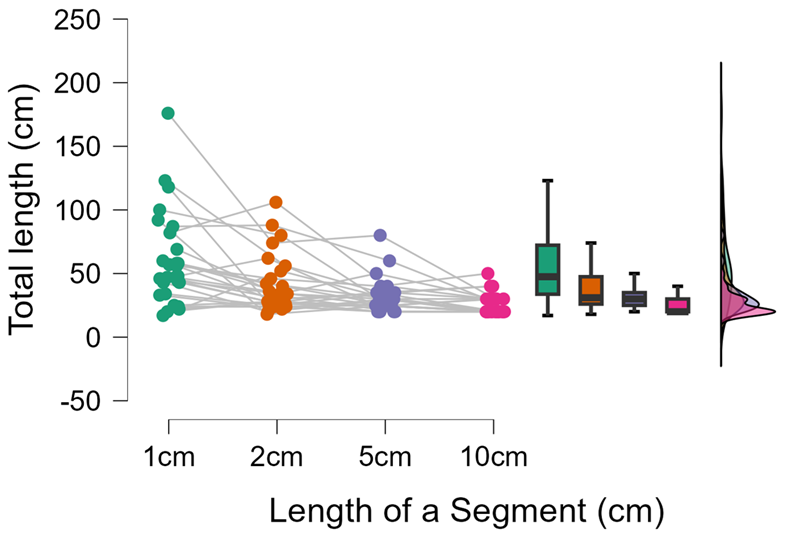
**

**Fig. 14** Distribution of participant responses for matching a reference length (20cm) of spaghetti using spaghetti pieces of 1, 2, 5 and 10cm cut spaghetti


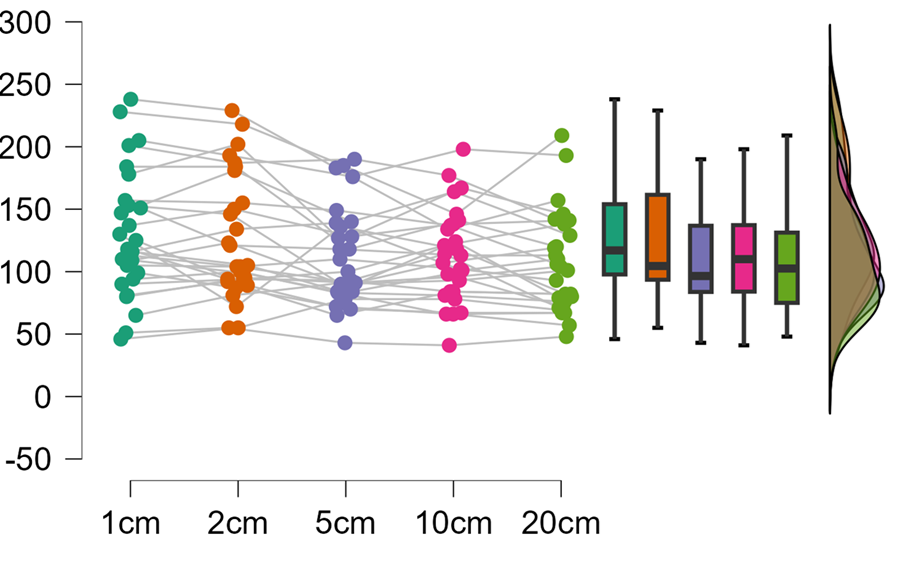


Fig 15. Distribution of responses for individual participant, estimating portion size of spaghetti (g) as a function of spaghetti segment length (cm).
